# Decision-making in surgical neonatal necrotizing enterocolitis

**DOI:** 10.4103/0971-9261.57701

**Published:** 2009

**Authors:** Mitul Parikh, Ram Samujh, Ravi Prakash Kanojia, K. L. N. Rao

**Affiliations:** Department of Pediatric Surgery, Post Graduate Institute of Medical Education and Research (PGIMER), Chandigarh, India

**Keywords:** Laboratory parameters, metabolic acidosis, necrotizing enterocolitis, neonate, peritoneal drain

## Abstract

**Aim:**

To know whether laboratory or clinical parameters can predict disease progression, need for laparotomy in patients managed with peritoneal drain and mortality in surgical neonatal necrotizing enterocolitis patients.

**Materials and Methods:**

The study was retrospectively carried out on 27 neonates over a period of one and a half year. All neonates who had surgical neonatal necrotizing enterocolitis in the form of bowel perforation, positive paracentesis, abdominal wall erythema and abdominal lump were included. Patients with Bell's stage I and those developing enterocolitis after surgery were excluded. The patients were evaluated with parameters, namely, clinical, laboratory and radiological. These included age and stage at presentation, primary symptom/sign at presentation with laboratory parameters of blood counts, pH, base deficit, platelet counts, electrolytes and random blood sugar levels. A comparison was done between survivors and nonsurvivors, patients with primary peritoneal drainage versus those requiring laparotomy after drain, Bell' stage II versus III patients and operated versus nonoperated patients. Statistical significance was observed in the above mentioned comparisons.

**Results:**

There were 22 male and 5 females patients with mean birth weight of 1.85 kg. Age at presentation ranged from 2 to 19 days, mean 9.25 days. Mortality was 37% (10/27). Majority of the stage II patients presented with feed intolerance and abdominal distension. The neonates with severe disease had abdominal distension with wall erythema. Sixty percent of the patients had shock at the time of admission. In the comparison of peritoneal drain only and patients with peritoneal drain followed by laparotomy patients, it was observed that neonates who were acidotic and had higher base deficit had more chances of requirement of laparotomy. They also had progressive fall in platelets counts. There was no difference in the birth weight, gestational age, total counts, serum electrolytes, blood sugar and other measured parameters; thus, these carry negligible predictive value to judge deteriorating neonate. In the remaining of the comparison, patients not presenting with shock were more likely to survive.

**Conclusion:**

In the present study, neonate with persistently low pH, higher base deficit and presentation with shock predicted need for laparotomy in drain managed patients as well as chances of survival.

## INTRODUCTION

Neonatal necrotizing enterocolitis (NNEC) is a major cause of neonatal abdominal emergency and carries a high mortality.[1] Various clinical and laboratory parameters have been extensively studied in the past and are related to the etiology and mortality of the disease.[2] We retrospectively evaluated 27 patients of NNEC admitted at out center. Patients who were managed surgically either by primary peritoneal drain and/or laparotomy formed the main basis of this study (surgical neonatal necrotizing enterocolitis). Various factors were studied for their significance in predicting progression of the disease, need for laparotomy in cases managed on peritoneal drainage and ultimate outcome of the patient. Identification of such factors in surgical NNEC patients adds a new dimension to the existing management protocol and helps in early recognition of a deteriorating patient.

## MATERIALS AND METHODS

The present study was retrospectively carried out at the department of Pediatric Surgery, PGIMER, Chandigarh, from January 2007 to June 2008. On an average, 45-50 neonates with NNEC are admitted per year. They are jointly managed by the neonatal and the pediatric surgical facilities at this center. Twenty-seven patients admitted during this time were included in the study. They had clinically proven diagnosis of NNEC and fulfilled one or more criteria of having surgical NNEC in the form of (1) clinical or radiological evidence of bowel perforation, (2) positive paracentesis, (3) abdominal wall erythema or edema indicating bowel gangrene, (4) presence of abdominal lump and (5) patients not responding to conservative management with falling general condition. Patients with Bell's stage I and those developing NNEC after surgery were excluded from the study.

The initial diagnosis of NNEC was mainly clinical, with abdominal distention and intolerance to feeds as major sign/symptoms; these findings coupled with information on various predisposing factors as prematurity, type of feed started, perinatal stress, associated conditions as congenital heart disease were also taken into the account.

The patients qualifying for the study were evaluated with parameters, namely, clinical, laboratory and radiological. The clinical parameters included gestational age, birth weight, modified Bell's stage of disease at initial presentation, presence of predisposing factor, type of feeds, primary symptom/sign at the time of admission and treatment response. The laboratory parameters included serial estimations of blood counts, electrolytes, blood pH, base deficit, blood culture, platelet counts and random blood sugar levels. The radiological examination was done with serial abdominal x-rays taken at 12-hour intervals.

For the purpose of description and analysis, a 4-way comparison was done from the data obtained. (1) Patients with primary peritoneal drainage (PPD) versus PPD followed by laparotomy, (2) survivors and nonsurvivors, (3) operated (laparotomy) versus nonoperated patients and (4) Bell's stage 2 versus stage 3 patients. The various said parameters were observed in these stratified groups and statistical significance was observed. Any factor that turned out to have statistical significance was presumed to be a predictor of the disease progression and would have helped in a more targeted disease intervention. Survivors were defined by patients who were alive at one month postdischarge from the hospital.

The surgical management given to the patients of NNEC with perforation was in the form of PPD with bilateral flank glove drain in all patients. Patients who showed definite signs of bowel gangrene, frank intestinal obstruction or having palpable lump with pneumoperitoneum were taken up primarily for laparotomy. Few patients required laparotomy after PPD as they either continued to have high drain output with fecal contents for more than 48 hours, persistent abdominal distention and intestinal obstruction with the infant not passing stools for more that 2 days or deterioration in their general condition despite being on medical management.

### Statistical Analysis

The data collected were analyzed using mean standard deviation (SD) for continuous data and percentages for categorical data. To compare two groups for categorical data, chi-square test was used, while for comparison of continuos data, Mann-Whitney test was used. p < 0.05 was considered statistically significant.

## RESULTS

The demographic counts are as follows. There were 22 males (81.5%) and 5 (18.5%) females. Birth weight (BW) was in the range 1-3 kg (mean 1.85 kg ± 0.71). Age at presentation ranged from 2 to 19 days (mean 9.25 days). Mean age at presentation for preterm patients was 9.3 days, while for term patients it was 9 days. Patients who were breast-fed presented with mean of 12 days and those who were top fed presented with mean of 7 days. Gestational age (GA) at the time of presentation was 27-42 weeks (mean 34.22 weeks).

**Figure 1:**
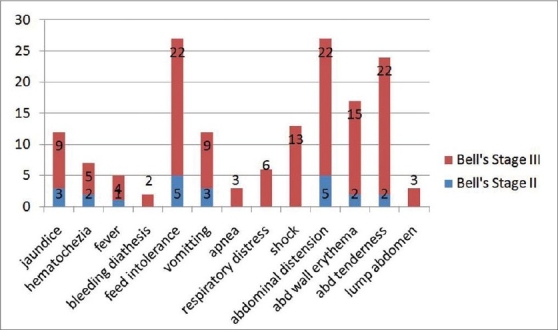
Bar graph showing the various kinds of presentations in patients with NNEC

Presentation of all patients is outlined in Figure 1, which also shows the breakup of presentation between Bells stage II and III disease. A majority of stage II patients had presented with feed intolerance and abdominal distension. The patients with severe disease predominantly had abdominal distension with wall erythema. Sixty percent of the patients had shock at the time of admission. Jaundice was an odd presenting feature in 41% of the patients.

**Figure 2:**
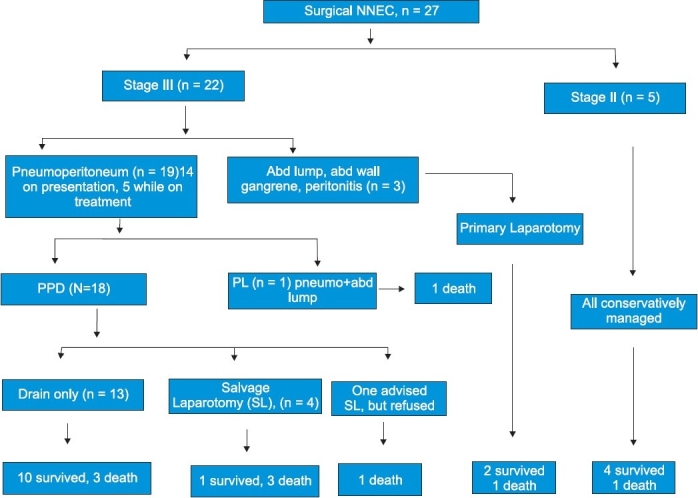
Flow chart showing the overall outcome and treatment done for the 27 patients

The flow chart in Figure 2 outlines the various treatments given to the patients with the indications. Five of 27 patients were treated conservatively, that is, without any drain. They initially showed features of abdominal wall erythema and distension, but since they were stable and had good general condition, they were observed to improve in 48 hours.

The clinical data of patients managed by PPD versus PPD followed by laparotomy was compared, to give and idea based on the parameters observed whether we can predict the need for laparotomy in patients managed by PPD. Patients who required laparotomy following peritoneal drainage [Table 1] had lower pH (*P* = 0.059) and higher base deficit (*P* = 0.039). They also had progressive fall in platelet counts, though it did not reach the statistical significance (*P* = 0.373) probably due to small sample size. There was no difference in birth weight, gestational age, total leukocyte count, Serum Na^+^ and random blood sugar levels, presence of shock at presentation, presence of positive blood culture or pattern of feeds before development of the disease.

**Table 1:** Comparison of parameters between those managed with peritoneal drain only to that requiring salvage laparotomy

	PD only (n = 18)	PD+LAP (n = 5)	*P*
Birth weight (kg)	1.75 ± 0.6	2.08 ± 0.25	0.275
Gestational age (wks)	34.08 ± 5	34.2 ± 1.78	0.656
Age at presentation (wks)	10.9 ± 4.7	5.8 ± 5.4	0.295
Serum Na^+^	138 ± 6.7	135 ± 8.6	0.622
Total leukocyte count	11437 ± 6389	12,500 ± 6349	0.256
Platelet concentration	124,153 ± 84,947	70,600 ± 64,534	0.373
pH	7.33 ± 0.06	7.22 ± 0.12	0.059
Base deficit	6.5 ± 2.9	11 ± 4.6	0.039
Random blood sugar	112 ± 28	111 ± 27	0.961
Positive blood C/S	7 (38.88%)	2 (40%)	1.000
Shock	6 (33.33%)	2 (40%)	1.000

We studied laboratory parameters to see whether there was difference in any parameter between operated and conservatively managed patients [Table 2]. In our study all patients who were operated were found to have either intestinal gangrene or perforation. Progressive increase in total leukocyte count, progressive fall in platelet counts and pH were observed in those who were operated, though there was no statistical significance. There was no significant difference in Serum Na^+^ and blood glucose levels between two groups.

**Table 2:** Comparison of those managed conservatively to those requiring laparotomy

Investigation	Operated (n = 8)	Nonoperated (n = 19)	*P*
Total leukocyte count	14,350 ± 3619	10,794 ± 5810	0.094
Platelet concentration	84,875 ± 25,831	127,736 ± 84,202	0.408
Serum Na^+^	132 ± 8.4	137 ± 7.1	0.221
pH	7.28 ± 0.09	7.33 ± 0.08	0.143
Base deficit	8.8 ± 5.1	6.5 ± 3.7	0.271
Random blood sugar	101 ± 31.7	106.9 ± 27	0.690
Survival	3 (37.5%)	14 (73.7%)	0.102

There were 17 survivors and 10 nonsurvivors in the studied patients. Table 3 shows various variables studied in the survivor and nonsurvivor groups irrespective of the type of treatment given. It can be summarized as follows: the survivors had higher mean birth weight (*P* = 0.650) and gestational age (*P* = 0.107) as compared with the nonsurvivors; however, no statistical significance was achieved. There was no marked difference between the mean platelet concentration and mean values of random blood sugar between the two groups. Sixty-six percent of the patients in the nonsurvivor group had presented with shock as compared with 40% in the survivor group. Statistical significance was noted in three variables, which included blood pH (*P* = 0.003), base deficit (*P* = 0.002) and number of patients presenting with shock (*P* = 0.001). Patients who died remained more acidotic and had higher base deficit as compared with the patients who survived. Almost all patients in the nonsurvivor group had presented with shock except for one patient (90% patients). Blood culture positivity for various organisms (*Escherichia coli* in the majority) was same in both groups.

**Table 3:** Comparison of various parameters between survivors and non survivors

	Survivor (n = 17)	Nonsurvivor (n = 10)	*P*
Birth weight (kg)	1.9 ± 0.67	1.81 ± 0.75	0.650
Gestational age (wks)	36.4 ± 4.8	33.2 ± 4.3	0.107
Total leukocyte count	11,558 ± 5772	12,340 ± 5093	0.514
Platelet concentration	128,235 ± 80,651	92,600 ± 58,273	0.216
pH	7.36 ± 0.04	7.24 ± 0.10	0.003
Base deficit	5.11 ± 2.52	10.8 ± 4.31	0.002
Random blood sugar	99.17 ± 27.26	115.4 ± 27.74	0.102
Shock	4 (23.5%)	9 (90%)	0.001
Positive blood C/S	6 (40%)	6 (66.7%)	0.400
STAGE - II	5 (100%)	0	0.124
III	12 (55%)	10 (45%)	

A cross-correlation between the severity of the disease (Bell's Stage) and the various laboratory parameters [Table 4] showed that platelets counts decreased significantly with the progression of the disease (*P* = 0.052 marginally not significant), and patients with stage III disease had higher base deficit and lower blood pH as compared with patients in stage II disease. There was no difference in total leukocyte counts, serum sodium (Serum Na^+^) and random blood sugar levels in patients with stage II and III disease. There was only one death in stage II group, whereas 46.5% patients with advanced disease died.

**Table 4:** Laboratory parameters to know the progression of the disease in NNEC

Investigation	Stage II (n = 5)	Stage III (n = 22)	*P*
Total leukocyte count	11,560 ± 2714	11,913 ± 5938	0.87
Platelet concentration	165,000 ± 69,493	103,545 ± 71,589	0.052
Na^+^	133 ± 5.6	136 ± 8.0	0.381
pH	7.38 ± 0.48	7.30 ± 0.09	0.017
Base deficit	4 ± 2.5	7.9 ± 4.2	0.040
Random blood sugar	88 ± 19.3	109 ± 28.59	0.126
Survived	5 (100%)	12 (54.5%)	0.124

## DISCUSSION

The advances in neonatology and modern neonatal intensive care units have resulted in better survival of both term and preterm infants. As a result of improved survival in the younger and lower birth weight babies, the incidence of NNEC has increased.[1,2] Despite several research efforts NNEC still remains a major cause of death among these surviving neonates. We conducted a retrospective study at our institute to know the factors predicting the progression of the disease, need for laparotomy in patients with pneumoperitoneum being managed with peritoneal drain and the mortality in surgical NNEC patients.

Approximately 27%-63%[3] cases with NNEC require surgical intervention in the form of either a peritoneal drain or a laparotomy, which is either primary or salvage. Extensive studies have been done in the past regarding the severity indices and monitoring of NNEC patients who are treated conservatively.[4] Very few studies have focused on the surgical NNEC patients.[5]

The favorable course in a patient with NNEC and perforation managed with PPD is to have a controlled fistula, which gradually resolves over a period of time. Several favorable clinical features in patients who are managed with PPD are adequate decompression of abdomen after drain placement, resumed bowel activity with the infant passing stools normally, minimal bilious nasogastric aspirates and stable or improving general condition.[3] On the other hand, patient may deteriorate and may have a persisting/increasing abdominal distention, high nasogastric output, intestinal obstruction and high fistula output. As a part of standard protocol, we tend to operate on these patients and majority of these patients will have intraoperative findings of multiple perforation, gangrene or small bowel cocoon formation.[6] There is another group of patients who will primarily require laparotomy without any drainage procedure. These patients are clinically obvious to have bowel gangrene, abdominal wall gangrene, palpable abdominal lump and present with severe sepsis and shock. The mortality is always high in the operated patients group;[7] this is evident from our study also where 5 of the total 8 patients (63.5%) who were operated died. The patients managed with PPD form a very salvagable group of patients who, if monitored properly, can be saved. The need for laparotomy in patients managed with PPD is mainly decided on frequent clinical and laboratory assessment. There are always borderline patients who make decision-making difficult, and this may cause delay in the laparotomy and further deterioration in the condition of the patient, ultimately affecting the survival of the neonate.[8] Various proposed laboratory measurements predictive of development and progression of NNEC are C-reactive protein, thrombocytopenia, blood lactate, metabolic acidosis, hyponatremia, a low absolute granulocyte count, leucopenia, high ratios of immature to total leukocyte counts (I/T ratios) and clotting abnormalities.[2] Our study has shown that some of these laboratory parameters help in predicting the need for laparotomy in patients managed with PPD but not responding to the treatment favorably.

Serial C-reactive protein levels monitoring can be useful to differentiate Bell's stage I from paralytic ileus or benign pneumatosis.[9] Persistently elevated levels may indicate the presence of stricture, abscess or gangrene. However, the fact that any patient with increasing septicemia and surgical stress of laparotomy will have elevated levels seems obvious.[10] C-reactive protein was not used as a factor in the presented group of patients.

Thrombocytopenia is a valid marker of late-onset sepsis with or without NNEC.[11] Decreasing platelet levels have been found to be associated with presence of gangrenous bowel and form a direct indicator for need of surgical intervention.[12] In the present series, we serially measured platelet counts in all patients with NNEC. Platelet counts were found to decrease significantly with the progression of disease. Those who required salvage laparotomy had significant decrease in the platelet counts, though it did not reach the statistical significant because of the small number of patients for the comparison [Table 2]. However, we could not study the behavior of the platelet counts before the development of the disease as most of the infants in our study were outborn (63%). We also could not identify the threshold level beyond which the prediction regarding the need for laparotomy (primary or salvage) could be made.

Blood lactate is known to increase as a result of tissue hypoperfusion and metabolic acidosis in neonates who are critically ill with sepsis.[2,13] This was also not taken in the present study as blood gas analysis will directly correlate with the lactate levels. Acidosis persisting even after adequate management is an indicator of progressive intestinal ischemic changes[14,15] and has been taken as an indication of surgery. A persistent pH of 7.2 or lower was regarded as an ominous prognostic sign of bowel necrosis.[16] Consistent with this we also found that the patients who died had lower mean blood pH value on serial estimations (7.24 ± 0.10, *P* = 0.003). Similarly, base deficit parallels the pH values and they were also found to be statistically significant (10.8 ± 4.31) for nonsurvivors as compared with (5.11 ± 2.52) survivors (*P* = 0.002). However, like platelet counts, we could not detect the threshold limit of the blood pH and base deficit values, which can help in decision making for the need for laparotomy in those who were being managed conservatively.

Total and differential counts are known to be predictor of mortality, specifically granulocyte count > 15000/mm^3^ was reported as poor prognostic indicator.[17] On the other hand, both neutropenia as well as increased total counts are seen in NNEC patients.[18] Higher counts are seen more in patients presenting with or developing perforation. We observed that patients who were operated had higher mean values of total leukocyte count as compared with the nonoperated group (14,350 ± 3619 vs 10,794 ± 5810), also the nonsurvivor group, which had predominantly operated patients had higher mean values (11,558 ± 5772 vs 12,340 ± 5093). Although these observations did not achieve statistical significance but it does indicate two things, one is the need for laparotomy and other is the increased occurrence of mortality.

Random blood sugar can be normal, elevated or decrease in NNEC.[19] This depends on the degree of sepsis the infant is having. Few studies in literature report that hypoglycemia is associated with infants who have cultures positive for Gram-negative bacilli, whereas normoglycemia is seen in patients who have mixed infections and hyperglycemia is seen in *E. coli* sepsis.[20] We observed that nonsurviving patients had higher mean RBS (115.4 ± 27.74 vs 99.17 ± 27.26). A majority in this group had blood culture positive to *E. coli* and yeast, whereas 11 of 17 patients who survived had sterile cultures and 6 had cultures positive to *Klebsiella* and *Enterococcus*. Overall blood culture positivity in the two groups was not statistically significant. With the above individual parameters studied it can be said that no one laboratory parameter alone is sufficient to predict the progression of the disease. Clincal assessment coupled with laboratory findings still remains paramount in diagnosis of disease progression.

The present study also compared various clinical parameters other than the laboratory parameters in patients who survived to those who died despite adequate treatment. The major observation was that those patients who survived had higher mean birth weight, gestational age and did not show falling platelets or total counts. However, these parameters were not statistically significant. On the other hand, three factors that were observed to have statistical significance were blood pH, base deficit and presentation with shock. Patients who died had consistently lower pH values and a higher base deficit as compared with those who survived even after correction as mentioned above. This is consistent with other similar studies.[5] This makes us assume that patients who remain acidotic even with adequate treatment will carry a higher chance of mortality, and hence these findings on serial estimations of blood gas analysis should alarm the managing surgeon of the possibility. The limitation to these conclusions, first we have a small sample size and the other is that a good number of patients who died were younger infants and were operated and the stress of surgery and the anesthesia will make the infant additionally acidotic. However, coupled with the observation that 3 of 13 patients who were managed on PPD only died and showed similar findings it consolidates the present presumption. Another factor was that patients who presented with shock, although initially managed and survived, died except one patient. Hence, presentation with shock was taken as a grave prognostic factor.

The study presented here had a limited sample size and perhaps the results obtained needs to be further validated with more number of patients. Among the identified risk factors for the mortality or the need for laparotomy in those managed with PPD only, we could not identify the threshold level, which can help in decision making. Although it does however give an insight into the predictive factors that may alert the managing surgeon or physician and help in early recognition of the deteriorating surgical NNEC patient. This will allow him to administer a specific directed treatment instead of only supportive management given to these patients.

## CONCLUSIONS

It is difficult to predict mortality on the basis of a single factor either clinical or a laboratory value. Lower blood pH and higher base deficit in surgical NNEC patients is a significant indicator of deteriorating patient and predicts mortality in this group of patients. However, this finding is corroborative with clinical assessment and needs validation with larger sample size. Even with great deal of available laboratory parameters, clinical assesment still remains the best bet in decision making in surgical NNEC patients.
